# Pilot study of a ketogenic diet in bipolar disorder: a process evaluation

**DOI:** 10.1186/s12888-025-06479-y

**Published:** 2025-01-21

**Authors:** Benjamin P. Rigby, Nicole Needham, Helen Grossi, Ivana Kamenska, Iain H. Campbell, Ben Meadowcroft, Frances Creasy, Cheryl Fisher, Pankaj Bahuguna, John Norrie, Gerard Thompson, Melissa C. Gibbs, Maja Mitchell-Grigorjeva, Ailsa McLellan, Tessa Moses, Karl Burgess, Rachel Brown, Michael J. Thrippleton, Harry Campbell, Daniel J. Smith, Sharon A. Simpson

**Affiliations:** 1 Population Health Sciences Institute, Newcastle University, Newcastle-upon-Tyne, UK; 2 MRC/CSO Social and Public Health Sciences Unit, School of Health and Wellbeing, University of Glasgow, Glasgow, UK; 3 Division of Psychiatry, Centre for Clinical Brain Sciences, Royal Infirmary of Edinburgh, Edinburgh, UK; 4 Department of Nutrition and Dietetics, Royal Hospital for Children and Young People, Edinburgh, UK; 5 Usher Institute, University of Edinburgh, Edinburgh, UK; 6 Psychiatry, NHS Lothian, Edinburgh, UK; 7 Health Economics and Health Technology Assessment, University of Glasgow, Glasgow, UK; 8 Centre for Clinical Brain Sciences, University of Edinburgh, Edinburgh, UK; 9 Nuffield Department of Clinical Neuroscience, University of Oxford, Oxford, UK; 10 Bipolar Scotland, Paisley, UK; 11 Department of Paediatric Neurology, Royal Hospital for Children and Young People, Edinburgh, UK; 12 EdinOmics Research Facility (RRID: SCR_021838), University of Edinburgh, Edinburgh, UK; 13 Centre for Engineering Biology, University of Edinburgh, Edinburgh, UK

**Keywords:** Bipolar type I or II disorders, Process evaluation, Neurophysiology, Metabolic psychiatry

## Abstract

**Background:**

Bipolar disorder is a serious mental illness, which requires new strategies for prevention and management. Recent evidence suggests that a ketogenic diet may be an effective intervention. This research aimed to explore the feasibility and acceptability of a ketogenic diet intervention for bipolar disorder, fidelity to its behavioural components and the experiences of the participants and research clinicians involved.

**Methods:**

A mixed-methods process evaluation was conducted. Semi-structured telephone interviews were carried out with 15 participants 1–2 months after completing a 6–8 week modified ketogenic diet intervention, and 4 research clinicians from the study team following the completion of data collection. Data were thematically analysed. Fidelity checklists completed by research dietitians were analysed using descriptive count and percentage statistics. Findings are reported post-hoc, following the analysis and publication of the main pilot study findings. Reporting was guided by the COREQ checklist.

**Results:**

Five themes were identified in the qualitative data: (1) ‘*Encouraging entry and supporting exit’* (e.g. recognising and managing participants’ varied motives and expectations, including around weight loss and symptom alleviation); (2) ‘*Challenging but potentially transformational*,’ which reflects that while it can be difficult to initiate and maintain a ketogenic diet day-to-day, many participants perceived physical and psychological benefits (e.g. significant weight loss, mood stability and enhanced ability to focus); (3) *‘Intervention facilitators*,’ including a range of behavioural (e.g. goal setting), social (e.g. family and dietitians) and technological (e.g. apps for monitoring) support mechanisms; (4) *‘Intervention barriers’* (e.g. dietary preferences, concerns about the diet and its impact, the testing burden and capacity of the delivery team); and (5) *‘The wider context’* (e.g. the cost of living and sociocultural expectations) was a crucial factor explaining differential experiences. Overall, descriptive analyses indicated moderate-to-good fidelity to the behaviour change components of the study.

**Conclusion:**

We provide novel insight into the experiences of people living with bipolar disorder initiating and following a ketogenic diet, as well as those of research clinicians who support the intervention. Future trials may benefit from increased clinical research capacity, better-defined entry and exit routes, additional interpersonal support, and greater understanding of how social and societal factors impact participation.

**Trial registration:**

Study registration number: ISRCTN61613198 (02/03/22).

**Supplementary Information:**

The online version contains supplementary material available at 10.1186/s12888-025-06479-y.

## Background

Bipolar disorder (types I and II) is a serious mental illness characterised by episodes of elevated mood and depression [1]. It has a lifetime risk of 1–2% [2] and is considered among the most challenging psychiatric disorders to manage [1]. Typically presenting during early adulthood, bipolar disorder is associated with significant economic and societal impact, comorbidities and high mortality [3, 4]. Life expectancy among people with this disorder may be up to 20 years less than the general population, with suicide and cardiovascular disease being leading contributors to excess deaths [1, 3]. However, current strategies for prevention and management of bipolar disorder are suboptimal and new interventions are required.

The ketogenic diet, premised on very low carbohydrate and very high fat intake [5], represents an intervention with emerging therapeutic potential across a range of psychiatric conditions, including bipolar disorder [6–9]. It is proposed that this diet may contribute to more stable energy production in the brain through the metabolism of ketones as an alternative energy source to glucose [10], thus overcoming issues of glucose metabolism and insulin resistance that are common among people with bipolar disorder [11–14]. However, despite promising case reports, there remain no randomised controlled trials (RCTs) examining the effect of a ketogenic diet in bipolar disorder.

A recent four-month pilot trial investigated the effects of a modified ketogenic diet on individuals with schizophrenia or bipolar disorder with existing metabolic abnormalities [9]. Among participants adherent to the diet, metabolic improvements were reported (e.g. reduced BMI and waist circumference measurements), as well as enhanced life satisfaction and sleep. In another recent pilot study, Needham et al. investigated the recruitment, feasibility and acceptability of a modified 6–8 week ketogenic diet in bipolar disorder among euthymic participants (i.e. those in a period of emotional homeostasis) [4, 5]. The key findings were that intervention recruitment and retention were feasible (20 of 27 participants recruited completed the 6–8 week diet), and that most participants reached and maintained ketosis during the study period (91% of all blood ketone readings suggested ketosis; all participants were in ketosis at least 70% of the time). Combined with promising outcomes data from this study (publication accepted [15]), these findings suggested that a full clinical trial is warranted [4, 15]. To complement this latter pilot study and inform the development of a future RCT (e.g. through understanding factors that influence study design or participant adherence to intervention), we conducted a mixed-methods process evaluation of the pilot post-intervention.

Process evaluations differ to outcome-focused research by exploring how interventions work in practice through increasing understanding of the implementation of interventions, the mechanisms of impact and the context that shapes these two factors [16]. Both quantitative and qualitative analyses can contribute to these aims [16, 17]. In the piloting phase, process evaluation helps understand fidelity, feasibility and acceptability, and optimise the future design and evaluation of full-scale trials [16]. Such considerations are particularly important in developing trials for population groups with severe mental illness, such as bipolar disorder, who may have complex barriers to research involvement [18]. For example, people with bipolar disorder are at risk of therapeutic misconception or optimistic bias when presented with research information [19], and often experience impaired concentration or memory [20]. This may affect the acceptability of and adherence to interventions such as the ketogenic diet, which is known to be challenging albeit achievable among adults [21–23]. Furthermore, the manifestations of bipolar disorder and its treatment can affect dietary intake, reinforcing the importance of examining acceptability. Periods of hypomania/mania can lead to erratic eating patterns, overeating or neglecting meals [24], while depressive episodes may reduce appetite and interest in food [25]. Additionally, some treatments, particularly mood stabilisers and atypical antipsychotics, may cause weight gain or metabolic changes, influencing food choices and appetite regulation [26]. Previous research has indicated that numerous components may be important to support adherence to and outcomes from dietary interventions for people with bipolar disorder, including goal setting, regular and tailored support from healthcare and allied professionals, and psychological therapies [27]. However, no studies have examined these and associated factors in relation to the ketogenic diet as a therapeutic intervention for bipolar disorder.

As part of a wider pilot trial [4, 15], this research aimed to further explore the feasibility and acceptability of the ketogenic diet intervention and elements of the study design, examine fidelity to the behavioural components of the intervention, and explore the experiences of participants and research clinicians involved in the research.

## Methods

The design of this research was pragmatic, though informed by behavioural theories. The writing of this manuscript was guided by the consolidated criteria for reporting qualitative research (COREQ) [28]; a completed version of the checklist is included in Additional file 1.

### The pilot study

This study was prospectively registered at isrctn.com under the registration number ISRCTN61613198 on 2nd March 2022.

A favourable ethical opinion was obtained for this study from the South East Scotland Research Ethics Committee 02 (approval number: 22/SS/0007) and it was approved by NHS Lothian Research and Development. The primary aim of the pilot study was to assess recruitment, acceptability and feasibility of a 6–8 week trial of a modified ketogenic diet for individuals with bipolar disorder. Secondary objectives were to assess the relationships of a biomechanical, metabolomic and brain imaging biomarkers with clinical and functional outcomes. In brief, the intervention comprised a diet with estimated energy requirements of approximately 60–75% from fat, 5–7% from carbohydrate and additional calories from protein [5]. Participants received tailored support from an experienced ketogenic dietitian, and had access to a psychiatrist on the research team if required. The support package was informed by the COM-B framework [29] and behaviour change theories [30, 31]. Twenty-seven euthymic individuals were recruited into the study. Full details about the study design, intervention components and participants are published elsewhere [4]; for ease of reference we provide a summary in Additional file 2. The clinical and functional outcomes will also be presented in a forthcoming paper, accepted for publication [15]. The remainder of this paper reports on sampling, recruitment, procedures and findings for this additional process evaluation component of the study.

### Participants

Fifteen of the 26 individuals recruited into the pilot study and who initiated the diet, and therefore already known to the study team, were purposively sampled, initially approached by the study coordinator via email, and agreed to be interviewed (a sixteenth participant originally agreed to participate but an interview was unable to be arranged). We aimed to achieve variation in participants’ age, gender, socioeconomic background and whether they completed the 6–8 week intervention. The Scottish Index of Multiple Deprivation (SIMD) was used as proxy measure of socioeconomic background, recording participants’ residential status on a scale of 1st decile (most deprived) to 10th decile (least deprived). Participant characteristics are reported in Table 1, which also provides comparison to the full pilot study sample.

**Table 1:** Participant characteristics (pilot study interviews)

Characteristics	Interviewees (*n* = 15)	Full pilot sample (*n* = 26)
Age	Mean = 48.5 years (range 37–54)	Mean = 45 years (range 26–54)
Sex	Female = 10 Male = 5	Female = 18 Male = 8
Place of residence (SIMD)	1st and 2nd deciles = 0 3rd and 4th deciles = 4 5th and 6th deciles = 1 7th and 8th deciles = 3 9th and 10th deciles = 7	1st and 2nd deciles = 0 3rd and 4th deciles = 5 5th and 6th deciles = 4 7th and 8th deciles = 7 9th and 10th deciles = 9 (Unclassified = 1)
Intervention completion	Discontinued = 1 Completed 6–8 weeks = 14	Discontinued = 6 Completed 6–8 weeks = 20

Table 1 shows that two-thirds of the participants were female and two-thirds were resident in areas in the four least deprived SIMD deciles. One of the 15 discontinued the diet before the 6–8 week intervention period was completed. Four research clinicians (all female) involved in the delivery of the intervention were also purposively recruited and agreed to be interviewed: two research psychiatrists (one of whom also acted as study coordinator) and two research dietitians. The descriptive quantitative analysis of the fidelity checklist comprised data from the 25 participants for whom at least one diet review meeting was recorded.

### Procedure

#### Qualitative component

One-off one-to-one semi-structured telephone interviews were conducted with study participants 1–2 months after completing the 6–8 week diet intervention, and research clinicians implementing the intervention following the completion of the intervention component of the study. The location of the researcher was a private office, while the location of participants at the time of interviews was unknown (likely varied). No third-parties were knowingly present at the time of interviews. The use of a semi-structured interview format allowed flexibility to ask additional questions based on the participants’ responses, and a set of prompt questions were used to stimulate discussion if needed [32]. Prior to the interviews, all participants were asked to read an information sheet and provide informed consent for this qualitative component of the study, which was additional to the consent already provided to take part in the dietary intervention. Participants were informed of the interviewer’s role and interest in the research. All interviews were conducted by BPR (a male university researcher, with extensive training and experience in qualitative methods), recorded on an encrypted electronic device, transcribed intelligent verbatim by a third party transcription service, and the transcripts were de-identified by BPR. Transcripts were supplemented by fieldnotes and post-interview summaries, which were not formally analysed. De-identification was performed manually line-by-line, and with guidance from the journal’s editorial team, removing potentially identifying information (e.g. substituting names with participant numbers; family relations, such as ‘my son/daughter’ being replaced with ‘my child’; or removal of specific locations). Member checking was not performed. The inclusion of quotations in the reporting of findings were reviewed by team members, which included checking for suitable de-identification. Interviews with the pilot participants lasted between 37 and 98 min. They were structured around the following topics: recruitment into the study; experiences of being a research participant; the diet; the support package; perceived outcomes; and areas for suggested development. Additional file 3 provides the interview guide. Interviews with research clinicians lasted between 22 and 45 min, and were structured around: general experiences; implementing the study; engagement with participants; and future directions (see Additional file 4 for the interview guide).

#### Quantitative component

Data were taken from the 104 diet review meetings in which fidelity checklists were completed in conversation between participants and a research dietitian. The checklists (see Additional file 5) were used by dietitians as self-report tools to ensure coverage of key behavioural strategies that underpinned the intervention: (i) providing information on the benefits of changing diet; (ii) goal setting and creating an action plan; (iii) social support; (iv) relapse prevention strategies; (v) reviewing the outcome of goals and plans; (vi) discussing (potential) barriers and how to overcome them; (vii) self-monitoring/feedback; (viii) discussing performance and providing feedback; (ix) discussing environmental restructuring; (x) discussing how doing well could be rewarded. No independent verification of fidelity checklists was performed. Hand-written checklists were compiled and cleaned by HG, IK and NN, then converted into digital MS Excel records by BPR.

### Analysis

Analyses were initially and primarily performed by BPR both alongside and after the examination of study outcomes; findings are reported post-hoc [16].

#### Qualitative analysis

Qualitative data were analysed using an inductive thematic approach based on Braun and Clarke’s original six-step method [33]. NVivo version 14 software (QSR International) was used to manage the data analysis process. Following data familiarisation (step 1), BPR conducted an initial open coding of all the transcripts. The resultant coding tree (containing both semantic and latent codes) was consolidated into an initial codebook by BPR, which was used by SAS to independently code two transcripts each from participants and healthcare professionals (step 2), after which discrepancies were discussed and the coding frame updated. The interpretation of the data were discussed between BPR and SAS, as well as through discussion with the wider team during meetings, before the final saturated code list was confirmed for further analyses. All transcripts were coded by BPR, and codes were collated into initial themes that directly responded to the specific questions of the process evaluation (i.e. matters of feasibility and acceptability), as well as data-driven themes (step 3). Themes were reviewed and refined iteratively through team discussion (steps 4–5), before write-up with illustrative quotations (step 6). No participant checking was performed.

#### Quantitative analysis

Quantitative data were initially analysed by BPR using descriptive counts and percentages to examine the proportion of meetings in which each behavioural strategy was covered and patterns in their use. Two sets of findings were generated, enabling examination of potential differences that might have influenced adherence and completion rates. First, an overall assessment of fidelity that included all 25 participants with completed checklists. Second, an adjusted assessment that only included data from participants that completed the diet at 6–8 weeks follow-up (*n* = 20). Data were not included in either analysis from any of the participants’ first meetings for two components, which by definition were not relevant to be discussed at that time point (reviewing goals and plans; and discussing performance). Fidelity anchor points were discussed and set by BPR and SAS, through reflection on previous research [34, 35]: moderate = 60–79%, good 80–100%. The interpretation of data were discussed at research team meetings.

## Results and discussion

### Qualitative findings

Five salient themes were identified in the qualitative data relating to recruitment, feasibility, acceptability and implementation of the ketogenic diet intervention. A summary of themes and their associated sub-themes is provided in Table 2.

**Table 2:** Summary of themes and sub-themes

Main theme	Subthemes
Encouraging entry and supporting exit	Addressing participant motives
	Setting clear expectations
	Providing exit routes
Challenging but potentially transformational	A big undertaking
	Perceived impact
Intervention facilitators	Behavioural support mechanisms
	Interpersonal support
	Teamwork
	Technology
Intervention barriers	The testing burden
	Capacity of the delivery team
	Dietary preferences
	Participants’ concerns
The wider context	Social pressures
	Keto in a cost-of-living crisis

### Theme 1 – encouraging entry and supporting exit

Findings from the pilot study suggested that recruitment and retention of participants was feasible [4]. This was reflected in the majority of experiences articulated through the interviews. While recruitment to this pilot was conducted primarily through Bipolar Scotland, the underlying mechanisms that encouraged entry into the study may be applicable in alternative contexts. For example, providing study-related resources so that people are *‘able to learn quite a lot about it before’* participation, through the website, *‘factsheets*,* and information about the study* (Participant 7).*’* Further, some participants valued hearing the experiences of a member of the research team (IHC) who has a diagnosis of bipolar disorder and follows a ketogenic diet.

He spoke, his presentation was excellent. And he lives with bipolar and he’s been on the ketogenic diet for about eight years and he could talk to the benefits that he felt. So it was very powerful (Participant 15).

#### Addressing participant motives

Participants entered the pilot study with various experiences of living with bipolar disorder. Some had been diagnosed many years ago, others as little as six-to-nine months prior to recruitment. Their ongoing treatment and management varied, as did their motives for participating. While participants sought alleviation of their symptoms, four wider and potentially stronger motives were identified. For some, the study represented hope. This was unsurprising given the challenge of managing this severe mental illness [1, 4].

I hoped that the keto diet would do something I haven’t been able to do on my own . I’d hoped that the keto diet would be a miraculous cure. I was looking for help or support to try and help me get better (Participant 8).

Other participants cited a desire to reduce drug-use and challenge *‘the current medical model for someone who has a diagnosis of bipolar [which] is to take medications and lower your expectations* (Participant 5).*’* Altruism was a third motive identified in the data.

I decided to participate because I felt like it was a very worthwhile thing to do … for other people and in the future. And for the knowledge that will be obtained from it, they’re actually treating people (Participant 1).

Eighteen of the 27 participants in the pilot study were overweight or obese [4], which is common among people with bipolar disorder [36]. For many, weight loss was a primary motive.

The anti-psychotic drugs make me crave sugar and my weight has just yo-yoed up and down. And I know that I should be eating low carb, it seems to work for me, it seems to really work for the weight loss. However, any time I’ve tried it before it just sends my mood loopy. I take more anti-psychotics, which makes me crave the sugar, and then it just goes round and round … as soon as I saw it, I was like, “I want to take part in this (Participant 13).”

#### Setting clear expectations

Given how challenging it can be to follow a ketogenic diet [5], it is important that potential participants are fully informed about the reality of participating in such a study. Several participants felt that the expectations were clear and realistic, which seemed in some cases to be linked to past experiences of medical intervention or research.

I’d been told before the appointments that’ll last this length of time. And I knew in advance about the once a week phone calls. And taking the blood samples and texting the results. Nothing took me by surprise … blood tests, I’ve had plenty of them. I took part in some research when I was first psychotic and even had the full brain scan at that point (Participant 13).

Meanwhile, others felt that expectations around the diet and testing regimens could be made clearer prior to engagement.

And I have to say, certain things, particularly in the first week, really threw me. I had no idea that’s what I was going to be involved in (Participant 15).

These experiences led the research clinicians to reflect and adjust their practice as the pilot continued. However, they also noted that baseline expectations were very high and may need to be managed carefully, as noted in previous research among people with bipolar disorder [37]. Furthermore, in pharmaceutical contexts, expectations of bipolar disorder treatment can significantly influence adherence to behaviours [38], and such factors should not be discounted in behavioural interventions.

I think we have discussed a bit as a team, and I think it would be really helpful to spend a little bit more time with people before they started the study to make sure that they were really clear exactly what the study would entail (Research Psychiatrist 1).

It was a different group with different needs, with different expectations, and the expectations, I would say, of the participants was extremely high. Because there’s so very little in terms of what can actually be done for bipolar disorder … the fact that looking to use this diet, I would say that the expectations of the participants were very high. Maybe that’s something that we need to be very aware of (Research Dietitian 1).

#### Providing exit routes

During the pilot, 77% (20 of 26) participants remained on the diet until 6–8 week follow-up [4]. One-third were reported to maintain their diet post cessation of the study period [15]. When interviewed one-to-two months post-follow-up, seven of 15 participants either stated that they remained on the diet, or expressed a desire to try the diet again. However, in most cases they reported concerns about the loss of support that came with being part of a research study, which may be compounded by the complexity of the ketogenic diet [5].

It’s not a simple thing and I was doing quite a lot of calculations and all that kind of stuff. And I suppose coming to the end of it and trying to find out how I can continue … because it really helped me, I felt so much better . I’m trying to find a way to do it. I’m not sure any of these ways [available online] are going to be as effective as the medical version, but I’ll keep experimenting. I think my understanding was that … staying on the version that I was on with the study isn’t advisable without supervision (Participant 6).

One of the research clinicians also reflected on the need for robust clinical protocols to support cessation or withdrawal from the dietary intervention.

I wasn’t maybe exactly sure how to explain to people how the withdrawal process worked or what next … and as you said, providing any aftercare reports. Some people just completely disengaged, so it was really difficult, we couldn’t even get in contact with them after they’d made that decision. And I suppose that’s another thing, should there be a process in place as to what to do if somebody completely disengages (Research Psychiatrist 1)?

#### General discussion of theme 1

The first theme highlighted the importance of supportive entry and exit routes from a ketogenic diet intervention for people with bipolar disorder. While we acknowledge that using stories from people with lived experience to aid recruitment may have the potential to bias study expectations or even outcomes (e.g. through a Hawthorne effect), we argue that these risks are outweighed by the important role lived experience plays in supporting people to engage with research. Given that self-efficacy among people with bipolar disorder may typically be low [39], such testimonies have the potential to enhance people’s belief in their abilities through encouragement and vicarious experiences [30], thus supporting them to engage with a novel and challenging activity, such as following a new diet. This approach may also support motivation through feelings of relatedness [40], and help set clear expectations of the study.

In future trials, it will be important to ensure that communications and recruitment strategies support a range of potential motives, which may extend beyond direct presentations of bipolar disorder. While significant weight loss has been observed in this pilot study and others [9, 15], the weight loss motive, as well as hope, should be addressed cautiously, given both the absence of high-quality RCT evidence of the effectiveness of interventions used in the management of obesity in people with bipolar disorder [36], and the risk of therapeutic misconception or optimistic bias in this population subgroup [19]. This reinforces the need for clear expectations around involvement in studies.

In terms of exit routes, anecdotal evidence suggests that trial participants experience various emotions when their involvement in a study ends, with isolation and uncertainty among other feelings being common [41]. Recognising this is particularly important for studies involving people with bipolar disorder, who may experience greater social isolation and loneliness compared to the general population [42, 43]. Findings from this study highlight the need to support participants’ motives both pre- and post-intervention, which includes setting clear expectations. This will enable participants to appreciate the potential challenges and rewards associated with interventions such as the ketogenic diet.

### Theme 2 – challenging but potentially transformational

In general, participants felt that being on the ketogenic diet was challenging. However, many of those interviewed considered the undertaking to be acceptable given the perceived benefits. For some, this was a transformational lived experience.

#### A big undertaking

Transitioning to and maintaining a ketogenic diet can be challenging for adults [21–23]. In this study, some participants recognised the value of being *‘recommended that I staged the introduction of ketogenic meals* (Participant 2),*’* and there is some evidence to support this from other clinical settings, which suggests better toleration [44]. For many, maintaining a daily blood ketone testing regimen, shopping and preparing food were perceived to be *‘very time consuming* (Participant 10)*’* and take considerable effort and preparation, particularly in the early stages of the intervention period, thus reflecting previous research.

It was quite tricky at first, but I quickly realised what was making it tricky was cooking for the other four [in the home]. And at first, I was like, I’ll try and incorporate meals that we can all eat. And then I was like no, that’s not going to work . So it was kind of like having to think about two lots of eating, what I was eating and what they were eating. But that got easier (Participant 19).

For several participants, this balancing of the diet with family commitments, including meal times, was just one of several ways in which the intervention was considered disruptive to everyday activities. Often, these perceptions related to the social aspect of eating.

Like how am I going to socialise with food so much a part of that in our culture? So I hadn’t somehow realised that that could be a barrier. And then carrying around the things that I needed with me if I was going somewhere. I decided not to go anywhere for the first couple of weeks to keep it easier. I think it might have been helpful to just know that, maybe you put this in place at a point where you can just take your time whilst you get into ketosis (Participant 1).

For a minority of participants, this sense of challenge may have been exacerbated due to cognitive difficulties.

I think probably the main barrier was lithium because measuring things and then going back to the charts … When I started having to calculate things, and I don’t necessarily think it would be difficult for an ordinary person, to be honest, but for me it was like, oh my God, this is like a major mental meltdown situation (Participant 14).

#### Perceived impact

The main outcome findings of this study are reported elsewhere [15]. However, all participants discussed their perceptions of the study’s impact, which often corroborated quantitative observations of weight loss and reduced mood lability, as well as results in other studies [9]. Additional benefits such as self-control and discipline were reported (Additional file 6 provides a full list of benefits reported during interviews).

I think even within the first two weeks, I felt like I got my brain back. It was an exceptionally stressful time, and it was nothing to do with the study. It just was in my life, like a lot of stress or highly stressful things to deal with. But I managed okay. I kept things to myself. I kept thinking to myself other times in my life this would’ve sent me under by now. These things would’ve sent me under, into some spiral of either high anxiety or depression (Participant 1).

As soon as I had used the keto for two or three days, I just felt more relaxed, more peaceful than I think I’ve ever felt in my life. And in that whole period since then, I don’t think I’ve experienced any fear or anxieties, hardly any agitations. This has always been a big problem, I seem to get very angry, so clearly that’s disappeared (Participant 14).

I’ve lost a stone and a half, probably more actually, nearer two stone. So that was a big thing for me (Participant 11).

Nevertheless, many participants also perceived a range of side-effects from the intervention that, as reported elsewhere, were generally mild and usually resolved with dietary adjustments [4]. For some, the lack of noticeable improvement in their mental or physical health status could be disappointing.

It’s likely out of frustration and that was just because I wasn’t seeing any positive changes as in weight loss, like proper changes … My mood would really dip when I did my blood tests, my finger prick and the ketones were high or the glucose … I was hypoglycaemic for a couple of times. But I got really down when I’d done everything and the ketones were high [but saw no results] (Participant 15).

Examples of negative experiences are also included in Additional file 6. To manage expectations, and overcome set-backs or side-effects, it is possible that the intensive tailored support received by participants in this pilot [4] may have triggered key mechanisms that facilitated adherence.

#### General discussion of theme 2

The findings set out in this second theme indicate that people with bipolar disorder may find the early stages of being on a ketogenic diet to be a considerable adjustment before familiarisation, particularly where family circumstances are considered. They suggest that additional support may be required in the initial few weeks, which are likely to be the most difficult [45], enabling participants to engage for long enough to experience perceived benefits. Attempts to settle into the ketogenic diet may be further inhibited by impaired concentration and memory, which are common among people with bipolar disorder [20]. However, similar symptoms may manifest with the onset of ‘keto flu’, which is a cluster of transient symptoms that can be reported as occurring within the early weeks of a ketogenic diet [46]. While it is not possible to discern the relative impact of these two possibilities in our study, it highlights a potential barrier.

Despite the presence of some challenges, the perceived positive impacts of this study are particularly noteworthy given the difficulty of managing bipolar disorder [1], and point toward the therapeutic potential of the ketogenic diet to address both a key characteristic of this severe mental illness (i.e. changes in mood) and commonly associated cardiometabolic risk factors in this population. The potentially transformational extent of these perceived and observed benefits for some participants may help explain why they undertook and continued a challenging lifestyle intervention, even after the study had finished. These perceived outcomes contributed strongly to its sense of acceptability, despite the challenges.

### Theme 3 – intervention facilitators

#### Behavioural support mechanisms

Previous research has tended to overlook the importance of different behaviour change techniques in dietary interventions [47]. Prompted by the intervention fidelity checklist (see Additional file 5 and quantitative findings for more detail), research clinicians supported participants to implement a range of behavioural support mechanisms tailored to participants’ needs, which they reported as being beneficial for adherence to the diet and testing regimen. These included action planning [48], behavioural prompts [49] and self-monitoring [50].

I’ve got a reminder in my work calendar, and then I’ve got a reminder in my personal calendar, and then if I’ve got a hectic day, I’m also setting a phone reminder so that an alert goes off (Participant 5).

I think it was just working out a good system, because we eventually got, like, a small diary, and then just wrote the recipes in it (Participant 7).

Another key behavioural strategy was setting and evaluating goals [51].

Every week we looked at what their goals were going to be for the next week, if we made changes, what kind of adverse events were they having (Research Dietitian 1)?

While importantly goal setting primarily supported adherence to the diet [52], the intervention may have also prompted some participants to reflect or act on bigger lifestyle goals. It is suggested that interventions for people with bipolar disorder need to extend beyond a narrow focus on adherence through traditional behaviour change techniques, to consider self-worth [53] and problem-solving that may underpin longer-term behavioural change [27]. For example, *‘my diet generally was absolutely shocking*,* so I figured it would encourage me at least to have some clear goal*,* even if it was from a physical health point of view* (Participant 11).*’* These wider goals may have been facilitated through behavioural and interpersonal support.

#### Interpersonal support

It was apparent that social support provided to participants by family, research clinicians involved in the study, and existing care teams was crucial, leaving some participants doubting the potential for the intervention to work outside the support of a research project.

And [my spouse] just helped me out, like she said, “if you’re having a brain meltdown just grab me, I’ll fix it.” So they’re a bit of an angel so I was very lucky. For a while we ate separate meals and then we both decided we were quite lonely so we started to combine them, which was much nicer (Participant 14).

I liked working with Research Coordinator and Research Dietitians, that was fantastic, great support from both of them … I don’t think people could really do this sensibly without that kind of support, it’s hard. And reflecting back on it. There are a few habits that I’ve gone back to (Participant 15).

This reflects research that argues providing consistent interpersonal support from family and delivery teams is pivotal for ketogenic diet interventions [54–56]. Some participants reported that close monitoring by the research clinicians gave them confidence in the study’s procedures.

I thought the support was really good. The dietitian was phoning you all the time and if you had questions, you could contact people, so you didn’t feel alone in a particular way (Participant 3).

Participants also commented on the contact time between themselves and the research clinicians (typically at least one diet review meeting per week). Most felt that this was adequate, while for some *‘it was probably better than I thought it would be* (Participant 4).*’* Most participants appreciated the responsiveness of the study team, their reassurance, and the way in which the diet, through the provision of recipes and resources, was tailored to their individual needs and preferences by one of the dietitians. This reflects previously observed benefits of tailored dietary information [57], despite concerns about a lack of practical examples of effective tailoring to dietary preferences in real-world weight loss contexts [58].

I don’t have issues about the amount of fat, but I do have some concerns about the kinds of fat. So the dietitian and I have worked on looking at this particular kind of fat and maybe I could switch it out for something (Participant 5).

While social support, broadly conceived, was evidently a strong facilitatory factor for participants and should be considered as a component of a future trial, it also played an important role among research clinicians delivering the intervention.

#### Teamwork

This pilot study had numerous components and involved a large team of people drawn from different academic and healthcare backgrounds. For example, psychiatry, radiology, neurology, dietetics and behavioural sciences. It was apparent that the cohesiveness of the research intervention team contributed to the feasibility of implementation. All research clinicians interviewed commented on the strength of the teamwork they experienced.

There were situations where I needed to contact The Study Coordinator, she responded quickly and we managed to resolve any issues or any further information … I did have someone else to direct any of the queries to and they were picked up and actioned by The Study Coordinator (Research Dietitian 2).

Underpinning this teamwork was a sense of effective communication and the use of modelling:

It was useful having the dietitian there as well because I talked it through with The Study Coordinator in the practice session, to sort of make sure that I was comfortable. There was a bit of delay between that session and seeing my first participant. The dietitian stepped in to do it again, so I got another viewing of actually how to [support] (Research Psychiatrist 2).

#### Technology

Different forms of technology were used during the study. These included text messaging, video calling and an app. Originally, all participants were supposed to have access to two apps through devices such as their mobile phones, to support logging and transfer of data from daily capillary readings and mood scales [4]. However, due to licensing issues, only participants recruited later into the study were able to access the Ketomojo app (for capillary readings). The use of the app was welcomed by both participants and research clinicians.

I think the app is essential for moving forward … That’s because all I did was pull up the device, draw the blood and then put it to one side and then carry on. If I had been able to log in the emotional stuff at that point it would have been good (Participant 5).

Rather than them text, they moved their data to Ketomojo. That was easy as well. Also, because if they forgot to send their data one day or they forgot to text it, we would text them to remind them to send it. Whereas if it was on the Ketomojo app, it was already there (Research Dietitian 1).

#### General discussion of theme 3

Our findings demonstrated that several key factors facilitate the implementation of a ketogenic diet for people living with bipolar disorder. Ensuring the intervention was underpinned by various behavioural mechanisms, and supported through interpersonal support for participants, teamwork among delivery staff and technological developments, all facilitated success.

The importance of effective collaborative efforts evident in our findings supports research that indicates implementation of complex, multispecialty clinical studies may require foundations of effective teamwork and communication strategies [59]. Shared study leadership and regular meetings between clinical and research staff, as used in this pilot, are considered important features of teamwork that can bridge and embrace different specialities [60].

Furthermore, the experiences of participants and research clinicians alike demonstrate how the use of technological developments can support with behaviour change techniques, such as prompts [49], but also contribute to the potential acceptability and feasibility of an intervention. Future trials may wish to explore the role of technological components more fully, including developments such as continuous ketone monitoring [61, 62]. The effective use of technology in the delivery of the intervention has the potential to reduce barriers such as the administrative burden on both participants and research clinicians.

### Theme 4 – intervention barriers

All participants and research clinicians commented on a range of potential barriers to the intervention. Broadly, these comprised those relating to implementation and delivery of the intervention, or constraints at the individual-level for participants. These were generally factors that could be managed during the intervention period, or have acceptable solutions for the future.

#### The testing burden

As part of the pilot, participants had initial and follow up meetings with a research dietitian and psychiatrist, as well as pre- and post-intervention blood tests and MRI scans. They were also asked to measure blood ketone and glucose levels and recorded elements of their mental state on a daily basis [4]. Many commented favourably on the comprehensiveness of these processes. However, some participants considered the testing regimen (i.e. both for outcomes and as part of the intervention) to be burdensome.

The very first [day of tests], I was exhausted. I’d come down from the north of Scotland and I didn’t quite know where I was going then. I eventually got myself there and I think there was a lot of questions. So it was quite heavy going to be honest. And then the next day I had the MRI and I didn’t sleep terribly well in the hotel. I fell asleep in the MRI (Participant 3).

This was also recognised by the research clinicians:

It could feel quite a lot seeing two people in one day, but I think it was perfectly manageable. I suppose, thinking from the participants’ point of view, it might have been too much. I tended to see them first, and so my bit was relatively more straightforward, but I know that they then give them lots of information with Research Dietitian 1. But she gave them chance to consolidate that (Research Psychiatrist 1).

The idea of a physical and emotional burden around the testing components of the study was also evident in relation to daily blood capillary readings for ketone and glucose levels. Some found the prospect of *‘finger pricking every day for two months*,* quite a lot to get your head round* (Participant 1),*’* while others felt a strong sense of responsibility to ensure that they were testing appropriately and providing good data, and welcomed individualised support as required.

I would worry that things had been recorded properly. Especially when I had to deal with two separate apps and for me it was so important that I did this properly. Like if I was going to do it, I was going to do it 100 per cent, as best as I could, you know what I mean? (Participant 1).

I was struggling with the Ketomojo device at the very start. I couldn’t seem to draw blood, I couldn’t seem to get the blood in the little thing, and it ended up The Study Coordinator was on the phone with me talking me through it (Participant 5).

#### Capacity of the delivery team

Three of the four research clinicians interviewed commented on difficulties they sometimes had with the study administration. Our findings highlighted ways that may support with these intervention-related tasks in future, for example the use of technology.

The paperwork burden is quite big, so you wonder about whether that could be done in a paper-light fashion so it could just be done online (Research Dietitian 2).

However, technology is no guarantee of a reduced administrative burden in clinical trials [63]. Some other challenges faced by the research clinicians were more measurement-related.

It was necessarily participant facing. It was just slightly tricky at points to arrange MRI scans at the research facility at the right time, because they only had fixed slots, and clearly participants have busy lives. That was quite often more difficult. Sometimes, when we were booking people in quite last minute, to find them hotels, the university service didn’t always have hotels available that quickly (Research Psychiatrist 1).

While impossible to offer a ‘walk-in’ service, these findings suggest that in any future larger trials, there needs to be sufficient capacity among the delivery team to undertake the administrative task load. Further, it may be necessary to increase the support available to participants, particularly dietetics.

If it’s a bigger number, you will need more dietitians… I mean, just to give you an example, when I said to you that we started seven people in one month, generally, within epilepsy, you would maybe start two or three a month. So starting seven in a month was hard, but it is doable. I think it would be important to know where our study participants were coming from, because our volunteers were all well [euthymic] people with bipolar disorder (Research Dietitian 1).

#### Dietary preferences

The experienced ketogenic dietitians worked closely with participants to tailor the intervention. This input ensured that, as far as was practicable, the prescribed recipes and suggestions reduced the risk of monotony associated with the diet [64], and aligned with participants’ dietary preferences. Unsurprisingly, some participants commented that there were aspects of the diet they did not like [65].

It was very hard to consume that amount of fat each day, that was another thing I wasn’t aware, that’s what the keto diet was all about. So, you know, you weigh out olive oil in the salad, you’re basically drinking olive oil at the end of the salad (Participant 15).

#### Participants’ concerns

It was apparent that participants had concerns about their involvement in this study, with some reporting in interviews that on occasions they experienced an elevated state of arousal or anxiety.

I think I was always a bit edgy about have I prepared. I was a bit hungry as well sometimes and it works as an appetite suppressant. I was a bit edgy about, just in general feeling that keto, keto, always talking about keto (Participant 4).

While concerns were generally perceived as mild and easily managed through self-regulation strategies, and the majority of participants experienced stable or improved mood lability, a minority of participants expressed feeling particularly anxious about the potential impact of the diet on their mood state, toward either hypomania/mania or depression, even if this did not occur.

One of the concerns I had was going into hypomania, just because of the change in my diet, big change and how that would impact my mood (Participant 12).

I suddenly got this anxiety that I was going to trigger a depressive episode, which I haven’t had for several years … The kids go to their dad’s about 40 per cent of the time, so I don’t have much backup (Participant 2).

#### General discussion of theme 4

Our findings reflect previous diabetes-related research that highlighted the potentially intensive and burdensome nature of daily self-monitoring of blood glucose and ketone levels [66, 67], as part of monitoring a ketogenic diet. Several participants commented on the importance of reassurance, education and a positive baseline testing experience for their engagement and adherence. In future trials, research clinicians should adopt a flexible approach to supporting emotional reactions to testing and continue to provide individualised assistance if required [66].

Further barriers related to dietary preferences, which research in paediatric groups has shown to have mixed impact on the ketogenic diet. For example, while existing preference for high-fat food types is not considered to influence adherence [68], overall dietary preferences are one of the strongest drivers of discontinuation [69]. There remains work to be done to better understand the role of preferences in adults with bipolar disorder, who can experience concomitant challenges of disordered eating.

At the time of entry into the study, participants were euthymic; only one participant experienced a period of hypomania and another a depressive episode during the intervention period, each lasting one month [4]. Nevertheless, these examples highlight the importance of understanding perceptions of mood among participants with bipolar disorder. In particular, while lifestyle changes have typically been studied for their therapeutic potential, little attention has been paid to how stressful or disruptive these may be. While all participants in the study had at least some contact with a psychiatrist on the study team [4], given that there is some evidence around the triggering effects of stress on depressive episodes in particular [70], it may be prudent for future studies to consider more regular psychological or psychiatric support and monitoring to identify and manage difficulties, which may arise from stress-related concerns about the diet.

### Theme 5 – the wider context

Our findings further demonstrated the benefit of process evaluation for identifying contextual factors associated with variation in participants’ experiences and outcomes in clinical studies [16]. Across the interviews, participants discussed various influences that either supported or constrained their engagement with, or adherence to, the intervention.

#### Social pressures

Most participants commented on the interaction between their individual involvement in the study and wider social environments. Two-thirds discussed how the study influenced their family life. For many this was manageable, but for those with complex family circumstances, this presented a considerable barrier to participation.

I’m a single parent of young children and I’m living on benefits. I’m doing voluntary work; I facilitate a self-help group … one of my children’s medical diagnosis played a role [in withdrawing from the study]. So, what happened was, staying overnight, having the brain scan and coming back, is really draining on my energy. I needed to organise childcare because I’m a single parent. It threw me slightly out of my routine. My child picked up on my stress and got quite disoriented, because we were doing things like Tuesday after school is my other child’s sports club … and this is what they’d got in their head. But this time I went via Lidl to get a particular Greek yoghurt that was important in the diet, and he just got so agitated, they nearly had a meltdown in the supermarket, because we’d gone off our normal (Participant 2).

The majority of participants mentioned how being on a ketogenic diet limited their abilities to socialise in ways they were accustomed. Some recalled needing to manage questions from others about their diet.

It was a wedding, a ladies’ evening, two graduations, so a formal dinner. And we talked about how to cope with that. And for the wedding, I took my little Pyrex dish of salad and avo[cado] and bacon … And I coped fine with it. I had to cope with questions about it but that was fine (Participant 15).

While in most cases participants adapted to the social aspect of the diet over time, and felt supported to handle such situations and perceived peoples’ reactions to be *‘understanding and cool with it* (Participant 6),*’* some found this created a talking point that raised the risk of unwanted disclosure about their condition.

One other thing is your disclosure status, because it’s quite a visible thing to be doing, to be eating so differently. And if people don’t know your diagnosis or why you’re doing it and stuff, that is just a level of complication, why are you doing it so strictly? … I kept myself to myself quite a bit, and the people that know my situation were the people that I tended to spend more time with. But with others I just used to say I’m on a diet (Participant 6).

The challenges of eating in social settings are an everyday reality for adults following a ketogenic diet; in many cases these are temporary as people learn to manage the challenges of the diet [71]. ‘Keto-friendly’ mitigation strategies, such as bringing one’s own meals to social events, are common.

Some participants also commented on how the diet interacted with their work lives. This relationship remains under-researched.

A lot of work commitments and things made parts of the diet difficult to do, but that was purely down to work more than anything else … the four o’clock [data] returns could be a bit tricky around work as well, but I understand why it was at that time of the day (Participant 11).

Most of these participants felt that being unemployed or able to work from home was a key factor in affording time to prepare meals and balance the requirements of the study around their other commitments. They recognised that others may be less fortunate, and there were several instances of people working away from home choosing to skip meals, struggling to find appropriate food in shops, or failing to return timely capillary blood readings.

#### Keto in a cost-of-living crisis

The pilot study took place toward the start of a period of significant economic uncertainty in the United Kingdom, characterised by high inflation and global unrest driving-up food and domestic energy prices [72]. It is notable that most participants in this study were from middle-to-higher socioeconomic backgrounds, according to SIMD data. This was reflected in many responses speculating about the potential impact of a ketogenic diet for those with less financial means. However, several participants also commented on their direct experiences of the costs associated with the intervention.

Yes, I think it [the bills] definitely increased. I had to buy stuff for me and then different stuff for my kids. It is quite a high protein diet so that is usually a bit more expensive than carbs and then fish, fruit and veg. Things like raspberries are quite expensive (Participant 4).

#### General discussion of theme 5

Previous research on the ketogenic diet for children demonstrated the impact on family members, for example through nonacceptance of the diet or disruption of family routines, including mealtimes [54, 73]. Our findings extend knowledge by uncovering similar patterns within the context of adults with bipolar disorder. Notably, our study suggests that participation in the intervention may adversely affect those who have caring roles within their families (e.g. for dependent children or older relatives). Similar pressures, at least in the early stages of the intervention, were also identified in other social settings.

Furthermore, and crucially, our findings should be viewed within the context of an ongoing cost-of-living crisis in Western societies. Generally, the ketogenic diet is considered to be expensive for consumers compared to other diets [54, 69]. Participants recognised this as a potential barrier to those struggling with the cost-of-living, as food prices increased by 9.2% in the year to December 2022 [74]. A concurrent decrease in meat, fish and dairy sales was observed across Scotland among the general population, due to rising costs [75]. Selecting such foods appropriate for the diet in this context highlights the imperative for intensive support from ketogenic dietitians, alongside other financial-related strategies to reimburse out-of-pocket expenses as provided in the present study (e.g. travel to testing sites), to ensure inclusivity of the intervention. Mitigating the effects of a cost-of-living crisis is crucial to avoid inequalities in recruitment, adherence and health outcomes. Further economic studies are required.

### Further suggestions for developments in future trials

Participants and research clinicians alike made numerous suggestions for developments that should be considered in related future research trials. Broadly, these related to behavioural, communication, delivery, material, psychological, social and technological factors (see Additional file 7). Some of these were addressed over the course of the pilot, for example enabling automated transfer of daily blood glucose readings to the research team [4]. The two most common suggestions from participants were as follows.

Seven of the 15 participants interviewed proposed the potential benefit of additional peer support for those living with this *‘isolating illness* (Participant 6).*’* This may range from pastoral support for those who are *‘struggling*,* [to] just pick up a phone and we can have a brief chat about it* (Participant 12),*’* to being motivationally *‘helpful as well*,* with making me keep at it* (Participant 4).*’* However, despite the potential popularity of peer support, the evidence to support this in interventions for people with severe mental illness remains equivocal [76, 77]. Any such support would need to be carefully considered in the context of potential participant blinding in trials.

Linked to the challenging nature of the intervention and the societal context discussed in this paper, the same number of participants also suggested future studies may consider additional material support, especially for those facing greater financial hardship or without suitable cookery skills:

I think the equity issue around this, and really reaching the people who, if it’s shown to work, it might benefit … And that’s not always the case for people and they don’t all have the same amount of support as I do. So I suppose for some people financial incentives might be a deal-breaker, and help buying the food or giving the food (Participant 6).

While direct financial aid and in-kind contribution, such as the provision of food parcels or pre-prepared meals on prescription [5], may support engagement among individuals from lower socioeconomic backgrounds (none of the participants interviewed were resident in the lowest SIMD quintile), people with bipolar disorder may be at greater risk of having difficulty cooking food for themselves [78]. This may be compounded by the complexities of preparing food in the ketogenic diet [5].

### Quantitative findings – fidelity checklists

Overall, there were 104 diet review meetings in which fidelity checklists were completed between participants and a research dietitian. Twenty-five of 26 participants who commenced the diet had at least one recorded meeting. Among these, 20 participants completed the diet for at least 6 weeks. Of these 20, most had five review meetings (*n* = 15), while three participants had four (including one who declined to work through the checklist in their first meeting); two participants had three and six meetings, respectively. The remaining five participants had either one or two review meetings each.

It was intended for most, not necessarily all, behavioural components to be addressed in a single meeting, while ensuring that all were considered over the duration of the intervention. Each was addressed, as intended, at least once with 17 of the 20 participants who completed the diet for at least 6 weeks. Most components were addressed on multiple occasions with all participants. Before and after adjustment for participant withdrawal, the overall percentage of meetings that addressed a majority of components was 77%.

**Fig. 1:**
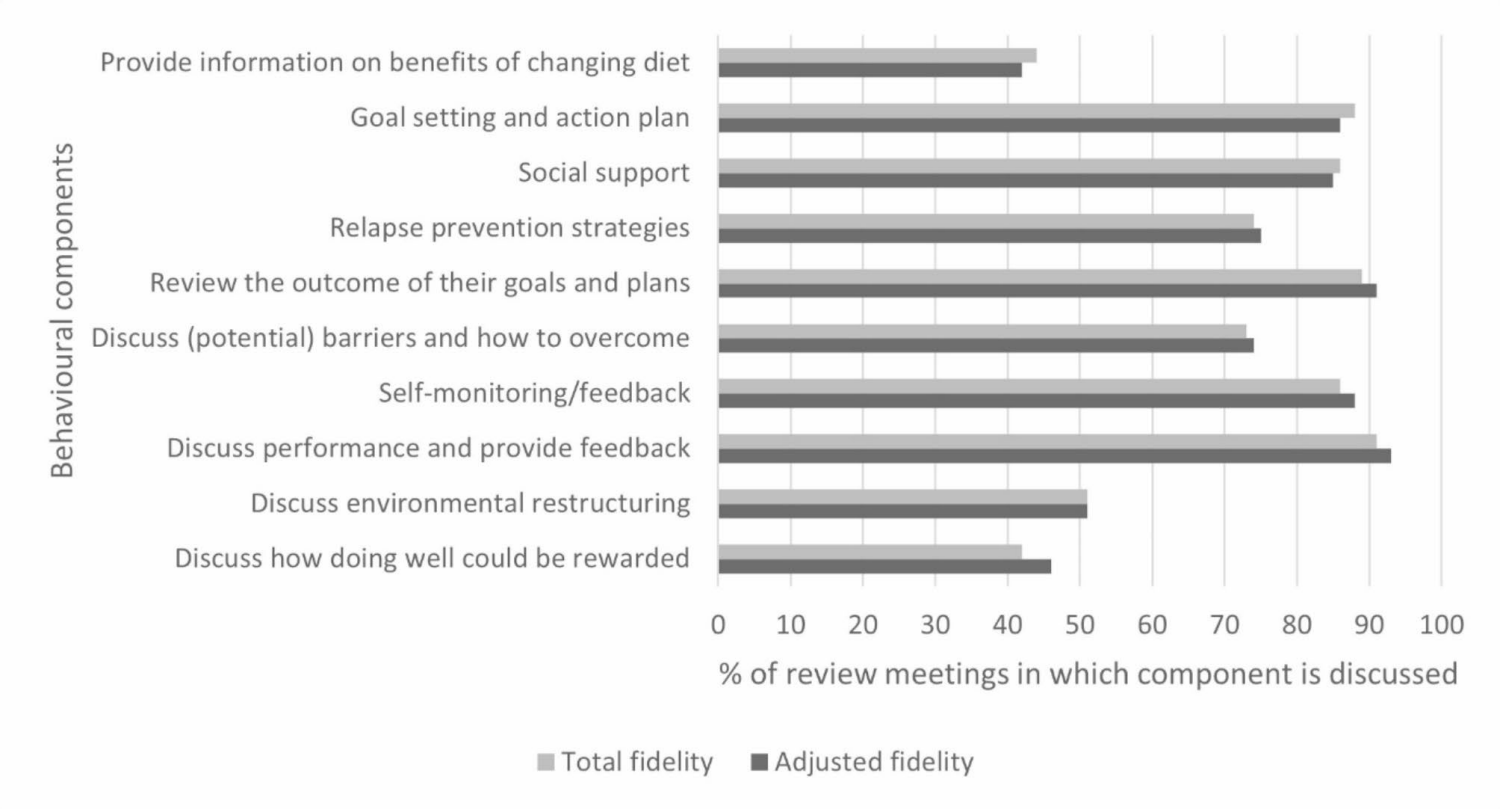
Fidelity of behavioural components implemented in diet review meetings

Figure 1 shows the percentage of diet review meetings in which the intended components were either discussed or implemented. Several behavioural components for the intervention were delivered with good fidelity. The most common component was discussing performance and providing feedback, which was documented in 93% of recorded checklists (91% non-adjusted). Setting and reviewing goals and action plans also featured prominently, perhaps reflecting its importance for people with bipolar disorder as it gives structure to their daily activities [27]. Meanwhile, three behavioural components were addressed less often. Providing information to participants on the benefits of changing their diet was discussed in 42% of meetings (44% non-adjusted); 70% of all instances in which this component was raised occurred during the first two review meetings across all participants. Discussing environmental restructuring occurred in 51% of meetings, and there did not appear to be any pattern to when this component was addressed. Finally, discussing how doing well could be rewarded was addressed in 46% of meetings (42% non-adjusted). Free text comments indicated that many participants felt that this was typically an unnecessary consideration, while one dietitian stated during their process evaluation interview that they *‘sometimes found that one a little bit tricky … a little but more of a struggle to think of potentially what the reward could be* (Research Dietitian 1).*’* This reflects previous research that suggests people find it difficult to consider non-food-based rewards for people on ketogenic diets [55]. It is proposed that participants may respond well to contingent rewards such as praise and encouragement in dietary interventions, which can support motivation and self-efficacy [79].

While many behaviour change techniques were implemented throughout the intervention period, the findings suggest that different techniques may have been used for different participants at various stages. This reflects the potential that different techniques may be more appropriate at certain times, and may better work for different people according to context and delivery method [80]. Further, different processes may need to be activated according to people’s readiness for dietary change [81]. This highlights the need to facilitate and understand participant expectations, and build rapport between participants and dietitians, for example through regular diet review meetings as in this pilot. It also indicates the importance of behavioural science support in future trials.

Overall, data indicated moderate-to-good fidelity to the behaviour change components of the intervention and have provided useful insights that can inform refinements to the behavioural strategies in future trials. Interviews with dietitians suggested that they both found the fidelity checklist a useful resource.

Because of fidelity questionnaire and because of the questionnaire that we had around things like adverse events … structured the interviews quite well. They were mostly all questions I would have asked anyway. So therefore being able to just sort of have that there, and you were recording as you were going along, then yeah, they were beneficial in that respect (Research Psychiatrist 2).

Checklists are an important strategy for examining fidelity in process evaluations and have been successfully used in dietary interventions [82, 83]. While the use of checklists in this study differed by being self-report tools to guide participant-dietitian interactions, our findings nevertheless reflected positive experiences associated with their use. There is emerging research to suggest that additional trialling of checklists at the onset of a study may improve application [84].

### Strengths and limitations of the study

This pilot study was among the first to demonstrate the feasibility, acceptability and safety of a ketogenic diet intervention for bipolar disorder [4, 15]. The process evaluation presented in this paper reinforces these observations. The intervention was theory-based and informed by current evidence regarding successful behaviour change techniques in dietary interventions. We used a rigorous multiple methods approach to data collection and analysis, and were able to triangulate data to strengthen the findings. Our research was further strengthened by the inclusion of 70% of all participants who continued the diet for 6 weeks and who completed follow-up assessments. We provide under-investigated insight into the experiences of people living with bipolar disorder initiating and following a ketogenic diet, as well as those of research clinicians who support this behavioural intervention.

However, our findings need to be considered in light of limitations in the study design and sampling. The post-hoc design of this study and short intervention period meant that findings and recommendations could not be addressed and further tested during the study. We were only able to arrange an interview with one participant who had withdrawn from the study and as such, may not have uncovered additional barriers and constraining contextual factors to engagement with a ketogenic diet. Relatedly, none of the participants in our sample were from the lowest socioeconomic areas (SIMD), where barriers may differ. Thus our findings may only reflect the conditions influencing participation among those resident in less deprived areas, where financial, social and intellectual capital may be more abundant and facilitative of engagement in challenging interventions such as this. The absence of these factors, known to disproportionately affect areas of lower socioeconomic status, are apparent in the barriers raised by our participants. Future studies should explore the potential need for additional support for these groups and others at risk of drop-out, to better understand contextual facilitators or constraints on their participation and adherence. While designed to primarily be a discussion aid, the use of self-report fidelity checklists risked introducing a Hawthorne effect, leading to potential overreporting of behaviourally-informed elements of participant-dietitian interactions. In terms of analysis, the strategy of having one predominant coder, opened the potential for bias. However, reflexive practices such as peer debriefing helped mitigate this issue. A final limitation related to our sample of research clinicians was that we only interviewed dietitians and psychiatrists. Further research is needed to understand the roles of other researchers involved in similar studies, such as imaging teams, given the potential importance of perceived positive baseline testing to the overall participant experience (i.e. feeling informed, cared for, and generally at ease). Additional areas of future work include further exploration of the motivation and engagement of the family in relation to providing social support, and mechanisms of dietary change.

## Conclusions

The findings provide valuable insight into the process of delivering and engaging in a ketogenic diet intervention for people living with bipolar disorder. In doing so, we highlight how challenging the ketogenic diet can be for some participants. However, for most people the intervention was considered feasible and acceptable and was delivered with good fidelity. Many participants reported a range of physical and psychological benefits, despite common side-effects, some less-positive experiences and some disruption to elements of daily and social living. To offset or overcome barriers such as dietary preferences, concerns about the diet and its possible impact, administrative burdens and limited implementation capacity, we advocate the incorporation of varied behavioural and social support strategies and technology assisted monitoring. Future trials may benefit from increased researcher/research team capacity, better-defined intervention entry and exit routes, additional interpersonal support, and a greater understanding of how social and societal factors impact participation among people with bipolar disorder, particularly among those most likely to be affected by these (i.e. those from more socioeconomically deprived backgrounds). This understanding can be facilitated by co-designing future clinical and behavioural interventions alongside people with lived experience of bipolar disorder.

## Electronic supplementary material

Below is the link to the electronic supplementary material.

Supplementary Material 1

Supplementary Material 2

Supplementary Material 3

Supplementary Material 4

Supplementary Material 5

Supplementary Material 6

Supplementary Material 7

## Data Availability

Data generated and analysed during the current study are not publicly available as explicit consent was not sought from participants, and privacy may be compromised.

## Abbreviations

BMI: Body mass index
RCT: Randomised controlled trial
SIMD: Scottish Index of Multiple Deprivation
