# Supplementary material for: Pilot study of a ketogenic diet in bipolar disorder: a process evaluation
Source: BMC Psychiatry. 2025 Jan 21;25:63. doi: 10.1186/s12888-025-06479-y (PMC11752864; doi:10.1186/s12888-025-06479-y)
Supplement: Supplementary file 1 — Supplementary Material 1 [file 12888_2025_6479_MOESM1_ESM.pdf]

Additional file 4: Interview topic guide (research clinicians)

Today, I am going to ask you to speak about your experiences of being involved in the study as a research clinician. I will ask you about your experiences of implementing the intervention and supporting participants. There are no right or wrong answers, and you should feel free to say as much or as little as you like. You do not have to provide an answer to a question if you do not want to. I may ask you to expand on your answers at times, but if you do not feel you have anything further to say, that is fine. We can take a break at any time, just let me know. Everything we discuss will be treated in confidence. If you want to finish the interview at any time, you can do so without needing to provide a reason for this.

Are you happy for me to start the recording?

General experiences

- Can you tell me a bit about your experience of working on this study? How did you find it?

Implementing the study

- Did you find the study paperwork easy to complete? If not, what issues were there?
- What were the barriers/facilitators for you to implementing the intervention? How did you try to overcome any barriers that you faced?
- What was your experience of using the Apps and other monitoring tools? Did you feel suitably equipped to support participants with these?
- Considering the fidelity checklist … was this useful?
- Which components of the intervention (including the behavioural strategies) did you implement more or less often?
- How did you try to incorporate the different behavioural strategies? Were any particularly easy/challenging to use?
- Were there aspects of the study that you struggled to implement as intended? If so, why?
- Did you make any changes to the way the intervention was delivered from the protocol?
- How did delivering the intervention and the different study components correspond to your usual working practices? Was it challenging?

Participants

- Through what media did the participants engage with you? What were the kinds of reasons they would get in touch?
- From your perspective what seemed to be the barriers/facilitators to participant engagement with the diet?

Moving forward

If we were to conduct this study in future…

- What additional support would research clinicians require?
- Do you have any suggestions as to how you might improve compliance with the diet?
- Do you have suggestions as to how we might increase recruitment into the study?
- Is there anything important that you would recommend that we change about the study or the intervention if we were to do it again, and why?
- Do you have any further thoughts or reflections about this study?
