# Supplementary material for: Pilot study of a ketogenic diet in bipolar disorder: a process evaluation
Source: BMC Psychiatry. 2025 Jan 21;25:63. doi: 10.1186/s12888-025-06479-y (PMC11752864; doi:10.1186/s12888-025-06479-y)
Supplement: Supplementary file 2 — Supplementary Material 2 [file 12888_2025_6479_MOESM2_ESM.pdf]

Additional file 2: Summary of the intervention delivery and implementation

1. Pre-recruitment appointments for information sharing and eligibility checking
2. Baseline appointments – information about establishing and maintaining a ketogenic diet, instructions on monitoring; taking of medical histories; metabolic and clinical measures were assessed; diagnostic interviews (completion of mental health measures)
3. Completion of a 3-day food diary and pre-ketogenic diet information sheet to aid planning and tailoring
4. Individual dietary prescriptions provided, commencement of dietary intervention
5. Support during the 6-8 week intervention period included weekly remote meetings with an experienced ketogenic dietitian (and more frequent appointments if required), to monitor progress, adjust prescriptions and provide general support. Psychiatry support was also available on demand
6. Participants were asked to provide daily blood capillary readings of glucose and ketones, as well as daily ecological momentary assessments of anxiety, mood, energy, impulsivity and speed of thought. Participants were also asked to complete 9 weeks of continuous accelerometery
7. Taking of medical histories, metabolic and clinical measures were assessed and diagnostic interviews (completion of mental health measures) were repeated at follow-up appointments (6 weeks was considered the necessary minimum for usable data collection in metabolomic and brain imagery analyses)

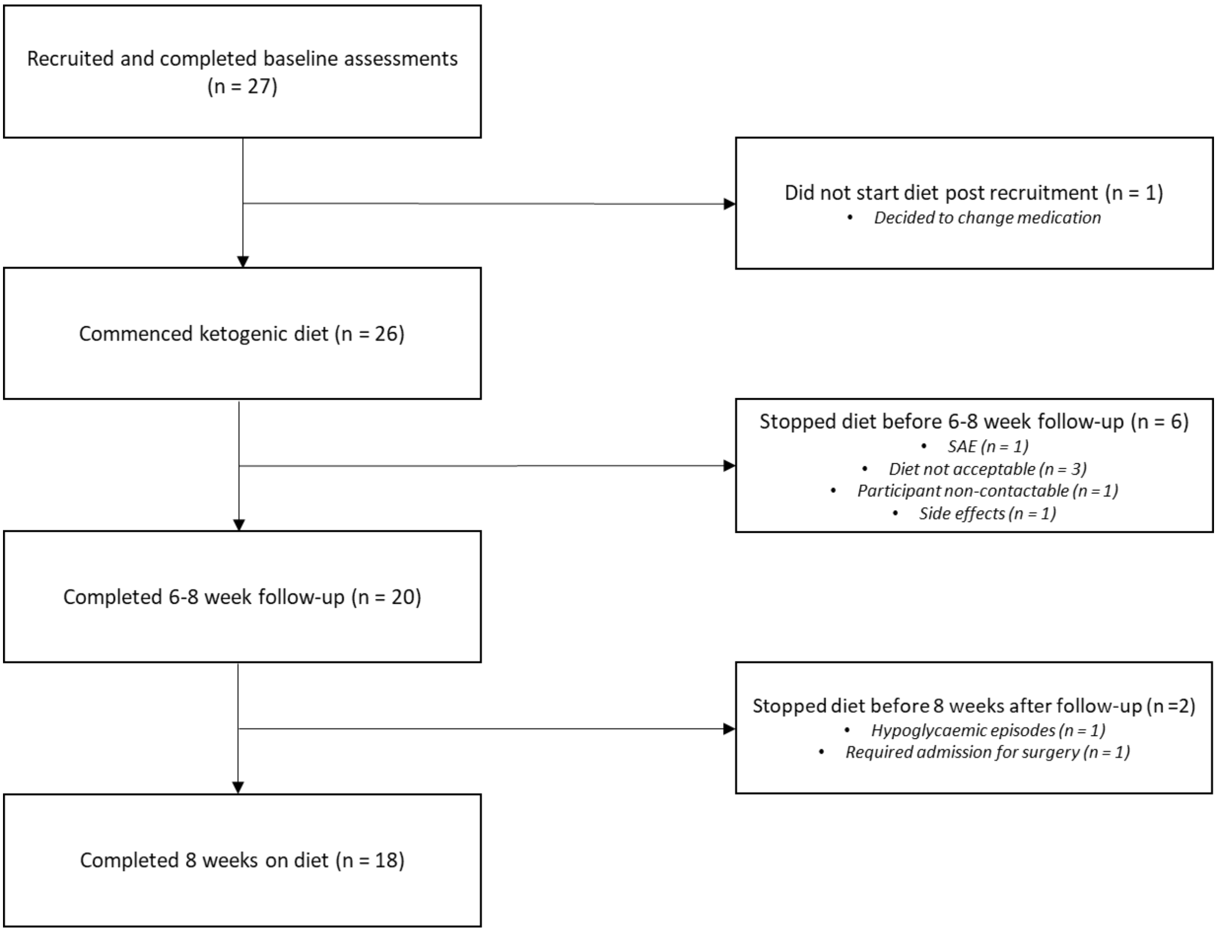

Flow chart illustrating the recruitment and completion rates of participants in the study (adapted from Needham *et al.* (2023)
