# Supplementary material for: Pilot study of a ketogenic diet in bipolar disorder: a process evaluation
Source: BMC Psychiatry. 2025 Jan 21;25:63. doi: 10.1186/s12888-025-06479-y (PMC11752864; doi:10.1186/s12888-025-06479-y)
Supplement: Supplementary file 3 — Supplementary Material 3 [file 12888_2025_6479_MOESM3_ESM.pdf]

Additional file 5: Intervention fidelity checklist

Session recording scoring sheet – Intervention sessions

Dietitian name:
Date:
Participant ID:

Element	1	0	Comments
Provide information on benefits of changing diet (e.g. the medical and personal benefits, thinking of the intervention not as a diet but a medication/treatment).			
Goal setting and a plan of action: (i.e. set or discuss/review goals to change behaviour).			
Social support (e.g. identify relevant family/friends who can offer support; other people also on keto diet e.g. via cooking classes).			
Relapse prevention strategies (e.g. ways to avoid eating certain foods; not putting themselves in certain situations).			
Review the outcome of their goals and plans (i.e. were the patient's achievement of their goals/progress discussed).			
Discuss (potential) barriers and how to overcome these (e.g. side effects, potential negative influence of family members).			
Self-monitoring/feedback (e.g. monitoring blood every day; monitoring mood).			

Discuss participant's performance (including success/failure) and provide feedback.			
Discuss environmental restructuring options (e.g. get rid of biscuits in the cupboard).			
Discuss how doing well could be rewarded (e.g. a trip to the cinema or other treat).			
General comments.			

1 = element conducted; 0 = element not conducted
