# Supplementary material for: Pilot study of a ketogenic diet in bipolar disorder: a process evaluation
Source: BMC Psychiatry. 2025 Jan 21;25:63. doi: 10.1186/s12888-025-06479-y (PMC11752864; doi:10.1186/s12888-025-06479-y)
Supplement: Supplementary file 4 — Supplementary Material 4 [file 12888_2025_6479_MOESM4_ESM.pdf]

Additional file 3: Interview topic guide (pilot participants)

Preamble

Today, I am going to ask you to tell us about your experiences of being involved in our study. I will ask you about your experiences of the ketogenic diet, the support you received, and how you felt about being a research participant. There are no right or wrong answers. Feel free to say as much or as little as you like, and remember that you do not have to provide an answer to a question if you do not want to. I may ask you to discuss things in a bit more detail, but if you do not feel you have anything further to say, that is fine. Some of the things we discuss today may be sensitive, and everything will be treated in confidence. We can take a break at any time, just let me know. If you want to finish the interview early, please say so, and be assured that you do not need to provide a reason for this.
Are you happy for me to start the recording?

Background and context

Lead question

- Can you tell me how long you have been living with bipolar disorder and how it has impacted you?

Taking part in the research: recruitment

Lead questions

- How did you find out about the study? What attracted you toward it?
- What information did you receive about the study? Was it helpful/what could be improved?

Prompts/probes

    - *Why did you decide to participate? Did you have any initial concerns?*

Taking part in research: your experience

Lead question

- How manageable was being a participant in this study?

Prompts/probes

    - *Did you face any barriers to taking part as in the study?*
    - *Were the expectations on your time realistic? How did the study fit around your existing daily life? How did it impact those around you (e.g. at home or at work)?*
    - *Did you feel able to keep up with the testing requirements (e.g. completing diaries or remembering to do blood tests)?*

Lead question

- Can you tell me a bit about the hospital visits – How was attending for MRI scans and venous blood tests before the study and at follow-up?

Lead question

I would like you to tell me a little bit about the different Apps and questionnaires that we used in this study (e.g. were they easy to use/not; can anything make them more useful).

Prompts/probes

    - *How did you find completing the mental health and health economics questionnaires before the study and at the follow-up? What did you think about being text daily for data (if used)?*
    - *What did you think about using the Ketomojo and Ilumivu apps (if used)?*

Lead questions

- What did you think about wearing an Actigraph device on your wrist?

- Throughout this study your diet and health were very closely monitored, how did this make you feel?
- How did taking part in this study interact with any ongoing management or treatment that you have for bipolar disorder?
- How could we better incentivise participants to take part in a study like this?

The diet

Lead questions

- What came to mind when you thought about making changes to your diet? Did the diet meet your initial expectations?
- What were the easier/harder aspects of the diet to put in place?
- What did you like and dislike about the ketogenic diet?

Prompts/probes

  - *How well did it fit into your existing eating patterns (e.g. were you able to retain the same mealtimes/did you find yourself snacking more)?*
  - *Was there anything that you did to help you 'stick to' the diet? How was this helpful/not?*

Support package

Lead question

- During the study, what type of help (info/resources/support) was the most helpful in relation to the diet? What was least helpful? Why?

Prompts/probes

  - *What do you think about the individualised recipe leaflets that you were provided?*
  - *Did you use the keto-calculator? If so, what did you think about this?*

Lead questions

Thinking about the research dietitian…

- Can you tell me a little bit about the initial meeting, how did that go?
- What support did you receive from them regarding the diet? Did you ask for anything specific?
- How did you choose to interact with them (e.g. telephone, email, face-to-face)? Do you have a preference, and if so, why? How much contact time do you think is about right?
- How did the support you received compare to the expectations that you had at the start of the diet?

Thinking about the study coordinator…

- Did you engage with them? If so, are you happy to tell me about that experience?
- What support did you receive from them regarding the study and diet?
- How much contact time with the study team do you think is about right?

Outcomes

Lead question

- Has the study helped you? How/why not?

Prompts/probes

  - *How have things changed for you as a result of the study? Have you seen changes in other parts of your life apart from diet?*
  - *Did you experience any negative side-effects from the diet? How did you manage these? How long did they last, were they tolerable?*

General

Lead questions

- What was the most important positive aspect of this experience for you?
- Is there anything important that you would recommend that we change about i) the study; or ii) the ketogenic diet intervention, if we were to do it again?
- Do you have any further thoughts or reflections about this study?
