# Supplementary material for: Pilot study of a ketogenic diet in bipolar disorder: a process evaluation
Source: BMC Psychiatry. 2025 Jan 21;25:63. doi: 10.1186/s12888-025-06479-y (PMC11752864; doi:10.1186/s12888-025-06479-y)
Supplement: Supplementary file 5 — Supplementary Material 5 [file 12888_2025_6479_MOESM5_ESM.pdf]

Additional file 7: Suggestions for developments in future trials

Category	Suggestions by participants	Suggestions by research clinicians
Behavioural	Incorporating a physical activity component alongside dietary component of the intervention.	-
Communication	Ensuring more detail about the realities of the diet and commitment are communicated to participants to set clear expectations.	
	Making more resources available in digital formats.	-
	Promoting the extensive of support for participants in the intervention at the point of recruitment.	-
	Providing individualised feedback to participants based on their test results.	-
	Simplifying written materials and resources associated with the dietary intervention and research project.	-
Delivery	Ensuring research clinicians have experience of this sort of intervention.	Ensuring sufficient capacity among research clinicians to reduce the participant caseload.
	Increasing the number of pre- and post-intervention testing sites (i.e. more hospitals), to reduce cost and disruption to participants.	Increasing numbers among research clinicians, including ensuring the clear delegation at times of staff absence.
Delivery (cont.)	Incorporating a longer trial or transition period to support participants to make the substantial dietary changes required.	Linking to participants' existing treatment and care teams, for example having access to medical records and communicating with primary care and psychiatry colleagues.

Category	Suggestions by participants	Suggestions by research clinicians
Material	Providing education or cooking lessons for preparing ketogenic meals.	Ensuring participant expenses reimbursement system is working efficiently.
	Providing support for the purchasing of appropriate foods, for example money, vouchers or direct provision of food parcels and prepared meals.	-
Psychological	Incorporating a psychological therapeutic component, such as talking therapy, alongside dietary component of the intervention.	-
Social	Incorporating a peer support mechanism, such as a buddy system, to support transition to diet, adherence and motivation.	Providing additional targeted support for most at risk individuals, such as newly diagnosed or those with complex family or work circumstances.
Technological	Providing more video call options for diet review meetings, as opposed to telephone reviews.	Converting materials and resources to digital copies, to reduce amount of paper and increase efficiency.
	Using apps and continued blood monitoring devices to automate the data collection process for mood questionnaires and ketone levels.	
