# Supplementary material for: Pilot study of a ketogenic diet in bipolar disorder: a process evaluation
Source: BMC Psychiatry. 2025 Jan 21;25:63. doi: 10.1186/s12888-025-06479-y (PMC11752864; doi:10.1186/s12888-025-06479-y)
Supplement: Supplementary file 6 — Supplementary Material 6 [file 12888_2025_6479_MOESM6_ESM.pdf]

Additional file 6: Perceived benefits and down-sides of the ketogenic diet reported during interviews

Perceived benefit	Illustrative quotation
Blood sugar levels	Plus, I didn't seem to have, like I say, I don't want to keep going on about my blood sugars, but I didn't seem to have spikes in my bloods, which I've experienced before the diet, and a bit afterwards as well (Participant 7).
Discipline and self-control	I think for a number of the participants, their dietary habits and even their meal patterns and things, they had been quite erratic. And even, you felt that that would give benefit, to be given that support and advice and then, hopefully, they would feel the benefit from the diet, as well as they started to make the changes. Because there were a few, yeah, definitely, as you say, that their patterns, the number of meals that they had, the regularity of them, wasn't, it wasn't normal. But I think, yeah, I think it helped to give them that support and direction to make changes around that as well (Research Dietitian 3).
Energy stability	I would say that I think it is initially lower energy, but ultimately more stable energy and no deficit in a sense, but also definitely, it doesn't, in a sense the low energy probably is a good thing if you're trying to avoid mania, or extreme mixed episodes. So I would say those things it does do (Participant 8).
Generally feeling healthier	Like I say, just the enlightenment of how much diet actually affects you and whether it's feeling better physically improves your mental health or whether it's specifically just affecting my mental health, I don't know (Participant 11).
Mood stability	I think even within the first two weeks, I felt like I got my brain back. It was an exceptionally stressful time, and it was nothing to do with the study. It just was in my life, like a lot of stress or highly stressful things to deal with. But I managed okay. I kept things to myself. I kept thinking to myself other times in my life this would've sent me under by now. These things would've sent me under, into some spiral of either high anxiety or depression (Participant 1).

**Perceived benefit**	**Illustrative quotation**
Self-efficacy	So yeah, it's given me confidence to go forward with it as sort of just a way of doing things. I've done all the hard work of working it all out (Participant 13).
Reduced alcohol consumption	And it's actually altered my entire approach to using alcohol other than as a nice drink with a meal, you know, it's a good thing. And it's very positive, yeah (Participant 15).
Reduced cravings	I found myself snacking much less because the fat immediately stopped my cravings, levelled me out, which is great. But obviously four meals a day I'm not used to that so I had a meal, lunch in the evening, which was new but I enjoyed it (Participant 14).
Weight loss	Well what I liked about it was the weight loss because I put on far too much weight ... I was like, this is sensible weight loss and look at this, I'm still eating the same plate of food and it didn't feel at any time that I was having to go hungry (Participant 13).

**Perceived down-side**	**Illustrative quotation**
Changes to bowel habits	Bowel issues definitely changed, I would say they definitely slowed down and so I take selenium husk probably once a day usually and that's fine, but if I don't do that then I definitely get constipation which I've never had before actually (Participant 14).
Feeling disheartened	No. well, the only time is that time, when I returned my stats that day, I was feeling awful, so I did say that (Participant 11).
Impact on exercise	I guess it impacted exercise to a certain extent. I think I still continued to exercise regardless of feeling a bit fatigued and low in energy because I do use it as a self-management tool (Participant 15).
No benefit	I do not believe that adopting a ketogenic diet for six to eight or ten weeks … I don't think you can verify if that helps in any way (Participant 5).
Sleep difficulties	There was sometimes that I couldn't sleep as well actually (Participant 6).
Smoking behaviour	I ended up smoking more which wasn't a great side effect (Participant 4).
