# Supplementary material for: Pilot study of a ketogenic diet in bipolar disorder: a process evaluation
Source: BMC Psychiatry. 2025 Jan 21;25:63. doi: 10.1186/s12888-025-06479-y (PMC11752864; doi:10.1186/s12888-025-06479-y)
Supplement: Supplementary file 7 — Supplementary Material 7 [file 12888_2025_6479_MOESM7_ESM.pdf]

Additional file 1: COREQ checklist

Section/topic	#	Checklist item	Reported in section(s)
**Domain 1: Research team and reflexivity**			
Personal characteristics	1	Interviewer/facilitator – *which author/s conducted the interview or focus group?*	Methods – procedure – qualitative component
	2	Credentials – *what were the researcher's credentials?*	Methods – procedure – qualitative component
	3	Occupation – *what was their occupation at the time of the study?*	Methods – procedure – qualitative component
	4	Gender – *was the researcher male or female?*	Methods – procedure – qualitative component
	5	Experience and training – *what experience or training did the researcher have?*	Methods – procedure – qualitative component
Relationship with participants	6	Relationship established – *was a relationship established prior to study commencement?*	Methods - participants
	7	Participant knowledge of the interviewer – *what did the participants know about the researcher (e.g. personal goals, reasons for doing the research)?*	Methods – procedure – qualitative component
	8	Interviewer characteristics – *what characteristics were reported about the interviewer/facilitator (e.g. bias, assumptions, reasons and interests in the research topic)?*	Methods – procedure – qualitative component
Theoretical framework	9	Methodological orientation and theory – *what methodological orientation was stated to underpin the study (e.g. grounded theory, discourse analysis, ethnography, phenomenology, content analysis)?*	Methods
Participant selection	10	Sampling – *how were participants selected (e.g. purposive, convenience, consecutive, snowball)?*	Methods - participants
	11	Method of approach – *how were the participants approached (e.g. face-to-face, telephone, mail, email)?*	Methods - participants
	12	Sample size – *how many participants were in the study?*	Methods - participants
	13	Non-participation – *how many people refused to participate or dropped out? Reasons?*	Methods - participants
Setting	14	Setting of data collection – *where was the data collected (e.g. home, clinic, workplace)?*	Methods – procedure – qualitative component

	15	Presence of non-participants – *was anyone else present besides the participants and researchers?*	Methods - participants
**Section/topic**	**#**	**Checklist item**	**Reported in section(s)**
Setting (cont.)	16	Description of the sample – *what were the important characteristics of the sample (e.g. demographic data, date)?*	Methods - participants
Data collection	17	Interview guide – *were questions, prompts, guides provided by the authors? Was it pilot tested?*	Methods – procedure – qualitative component; Additional file 3
	18	Repeat interviews – *were repeat interviews carried out? If yes, how many?*	Methods – procedure – qualitative component
	19	Audio/visual recording – *did the research use audio or visual recording to collect the data?*	Methods – procedure – qualitative component
	20	Field notes – *were field notes made during and/or after the interview or focus group?*	Methods – procedure – qualitative component
	21	Duration – *what was the duration of the interviews and focus groups?*	Methods – procedure – qualitative component
	22	Data saturation – *was data saturation discussed?*	Methods – analysis – qualitative analysis
	23	Transcripts returned – *were transcripts returned to participants for comment and/or correction?*	Methods – procedure – qualitative component
**Domain 3: analysis and findings**			
Data analysis	24	Number of data coders – *how many data coders coded the data?*	Methods – analysis – qualitative analysis
	25	Description of the coding tree – *did authors provide a description of the coding tree?*	Methods – analysis – qualitative analysis
	26	Derivation of themes – *were themes identified in advance or derived from the data?*	Methods – analysis – qualitative analysis
	27	Software – *what software, if applicable, was used to manage the data?*	Methods – analysis – qualitative analysis
	28	Participant checking – *did participants provide feedback on the findings?*	Methods – analysis – qualitative analysis

Reporting	29	Quotations presented – *were participant quotations presented to illustrate themes/findings? Was each quotation identified (e.g. participant number)?*	Results and discussion
	30	Data and findings consistent - *was there consistency between the data presented and the findings?*	Results and discussion
	31	Clarity of major themes – *were major themes clearly presented in the findings?*	Results and discussion
	32	Clarity of minor themes – *is there a description of diverse cases or discussion of minor themes?*	Results and discussion
